# Supplementary material for: The Ultimate List of the Most Frightening and Disgusting Animals: Negative Emotions Elicited by Animals in Central European Respondents
Source: Animals (Basel). 2021 Mar 9;11(3):747. doi: 10.3390/ani11030747 (PMC7999229; doi:10.3390/ani11030747)
Supplement: Supplementary file 1 [file animals-11-00747-s001.zip › FigureS1.pdf]

AFQ

Listed below are 13 animal groups. Using the following scoring scale, please rate the intensity of your fear elicited by each of the listed animals.

1	2	3	4	5	6	7
**NO FEAR**	**VERY LITTLE FEAR**	**A LITTLE FEAR**	**MEDIUM FEAR**	**MUCH FEAR**	**VERY MUCH FEAR**	**EXTREME FEAR**

1. Worms
2. Mice and rats
3. Intestinal worms
4. Spiders
5. Snakes
6. Unfamiliar dogs
7. Stinging insect
8. Big carnivorans
9. Crocodiles
10. Birds of prey
11. Sharks
12. Large ungulates
13. Blood-sucking parasites

Do you have any negative experience with an animal? If yes, which animal was it? Please shortly describe the situation.

Does your fear of animals limit you in your personal or professional life in any way? If yes, which animals or situations cause it?

FigureS1: Animal Fear Questionnaire (AFQ), a self-reported scale measuring non-specific fear of animals.
