# Supplementary figures and images for: The Ultimate List of the Most Frightening and Disgusting Animals: Negative Emotions Elicited by Animals in Central European Respondents
Source: Animals (Basel). 2021 Mar 9;11(3):747. doi: 10.3390/ani11030747 (PMC7999229; doi:10.3390/ani11030747)

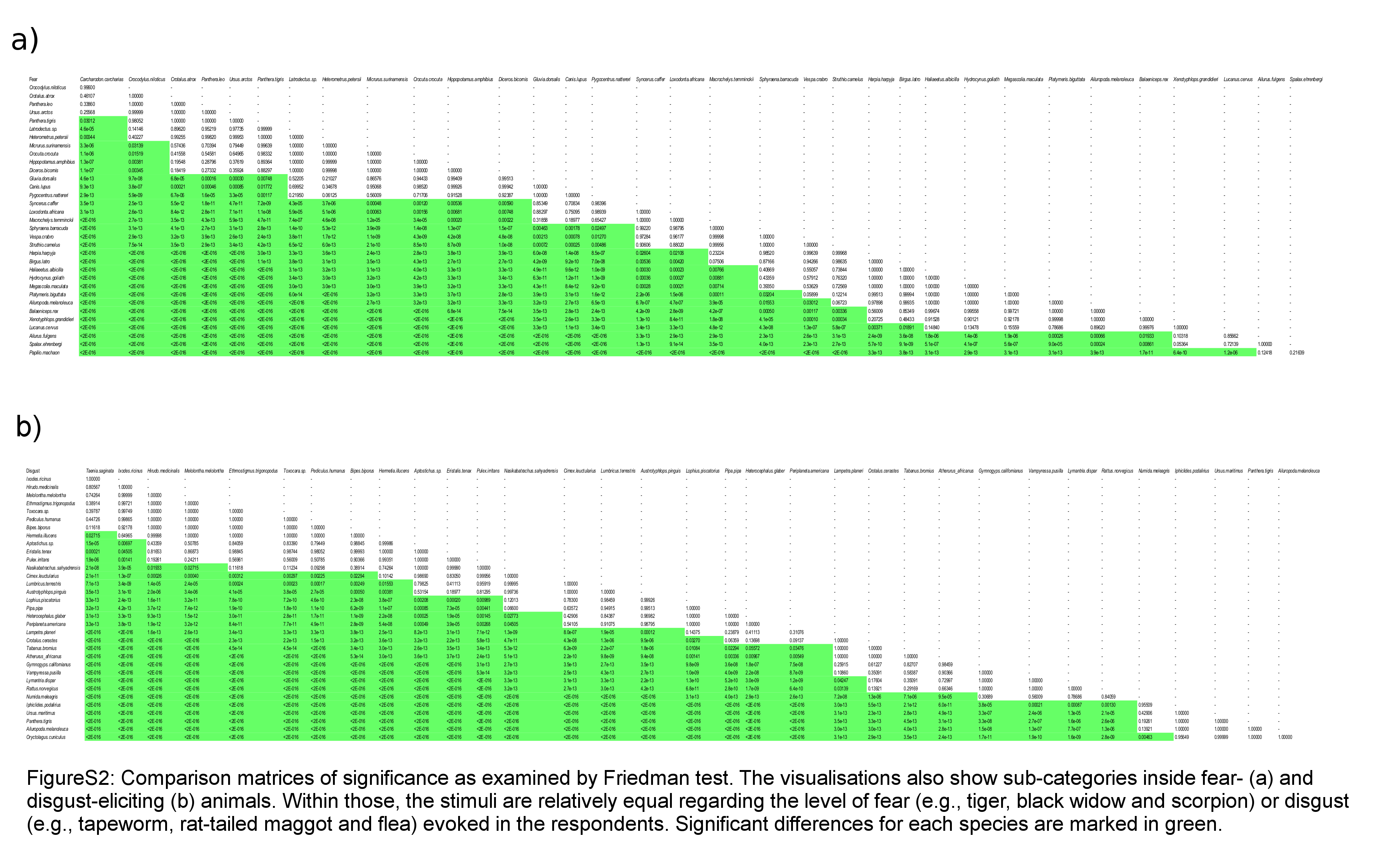

Supplement: Supplementary file 1 [file animals-11-00747-s001.zip › FigureS2.tif]

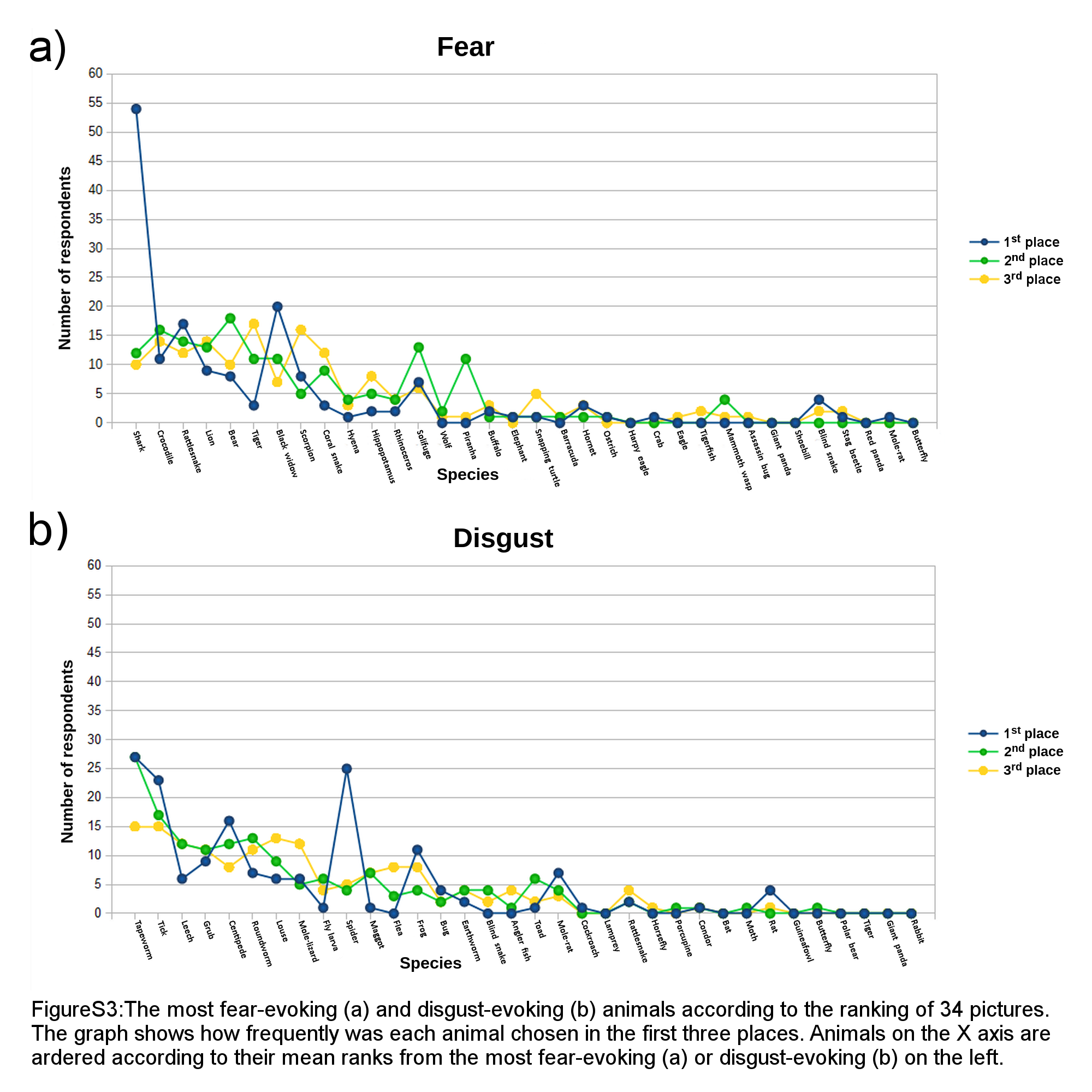

Supplement: Supplementary file 1 [file animals-11-00747-s001.zip › FigureS3.tif]
